# Neoadjuvant chemotherapy for Patients with advanced epithelial ovarian cancer: A Meta-Analysis

**DOI:** 10.1038/srep35914

**Published:** 2016-11-02

**Authors:** Long-Jia Zeng, Chun-Lin Xiang, Yi-Zhen Gong, Yan Kuang, Fang-Fang Lu, Su-Yi Yi, Yue Zhang, Meng Liao

**Affiliations:** 1 Department of Gynaecology and Obstetrics, the First Affiliated Hospital of Guangxi Medical University, Nanning, Guangxi, P. R. China; 2 The First Clinical Medical College of Guangxi Medical University, Nanning, Guangxi, China; 3 Department of Evidence-based Medicine, the First Affiliated Hospital of Guangxi Medical University, Nanning, Guangxi, P. R. China

## Abstract

The value of neoadjuvant chemotherapy (NAC) has not yet been fully defined. We aimed to systematically evaluate the influence of neoadjuvant chemotherapy (NAC) on survival and complete cytoreduction after debulking surgery in advanced epithelial ovarian cancer (AEOC) patients. We searched PubMed, Embase, and the Cochrane Central Register of Controlled Trials for the randomized controlled trials (RCTs) comparing NAC and primary debulking surgery (PDS) in AEOC patients. The last search date is February 25, 2016. Cochrane systematic evaluation was used to evaluate bias risk of included studies. RevMan 5.3 software was used for statistical analysis. A total of 4 RCTs involving 1922 patients were included. Compared with PDS, NAC may contribute to the completeness of debulking removal [no residual disease (RR: 2.37; 95%CI: 1.94–2.91; P<0.00001), residual disease ≤1 cm (RR: 1.28; 95%CI: 1.04–1.57; P = 0.02), optimal cytoreduction rate (RR: 1.76; 95%CI: 1.57–1.98; P<0.00001)], but there were no significant differences in both groups with regard to overall survival (HR: 0.94; 95%Cl: 0.81–1.08; P = 0.38) and progression-free survival (HR: 0.89; 95%Cl: 0.77–1.03; P = 0.12). This meta-analysis indicates that the higher rate of optimal debulking made NAC more favorable as a treatment option for AEOC patients with non-inferior survival compared with PDS.

The standard of care for advanced epithelial ovarian cancer (AEOC) has been primary cytoreductive surgery (PDS) followed by systemic chemotherapy. Neoadjuvant chemotherapy (NAC) followed by interval debulking surgery (IDS) has recently been put forth, as an acceptable standard of care for AEOC stimulated by the results of numerous studies^1^,^2^. Despite being recommended by 2015 NCCN guideline for patients with bulky stage III/IV disease, who are diagnosed by fine-needle aspiration (FNA), biopsy, paracentesis, or poor surgical candidates due to high-risk comorbidity conditions or disease factors^3^, the utility of NAC remains in dispute, especially whether NAC improves the prognosis of AEOC and which patients benefit most from NAC^1^,^2^,^4^,^5^,^6^,^7^.

To our knowledge, 4 large randomized controlled trials have been conducted^1^,^2^,^4^,^5^, assessing the benefits of NAC for AEOC, however, their results are conflicting. Kehoe *et al*.^1^ published a randomized phase III trial comparing NAC versus PDS in patients with AEOC, which showed equivalent survival in these patients, though NAC was associated with fewer complications and lower treatment-related mortality after IDS. These findings correspond with the data from Vergote *et al*.^2^, but not of Van Der Burg *et al*.^5^, in which patients with AEOC achieved longer survival with NAC followed by IDS compared to PDS.

Several meta-analysises, based on retrospective studies, have been performed^8^,^9^. The present meta-analysis was restricted to RCTs in order to eliminate selection bias, and overcome the limitation of retrospective studies.

## Methods

### Data sources and search strategy

PubMed, Embase, and the Cochrane Central Register of Controlled Trials databases were comprehensively and systematically searched from inception to February 25, 2016 without language restrictions. The search was limited to RCTs that compared NAC versus PDS for patients with International Federation of Gynecology and Obstetrics (FIGO) stage IIB-IV epithelial ovarian cancer. The search terms included “ovarian cancer”, “ovarian carcinoma”, “ovarian tumor”, “neoadjuvant chemotherapy”, “preoperative chemotherapy”, and “cytoreductive surgery”. References of relevant literature were also manually screened.

### Study selection and eligibility criteria

Articles were retained if they fulfilled the following predefined criteria: (1) subjects were patients whose pathological diagnosis was AEOC, with FIGO stage IIB-IV; (2) interventions were platinum-based NAC followed by IDS and chemotherapy OR PDS followed by NAC then IDS followed by chemotherapy, compared with PDS followed by platinum-based chemotherapy; (3) type of study was RCT. Articles were excluded if they were review articles or ongoing studies, conference abstracts, or without survival data. Two authors (LJ Zeng and CL Xiang) evaluated all articles to verify the inclusion and exclusion criteria independently. Differences of opinion were resolved by consensus after consultation between the two reviewers.

### Data extraction and quality assessment

Two independent reviewers (LJ Zeng and CL Xiang) performed data extraction (first author, year of publication, sample size, age, FIGO stage, pathology, histological grade, intervention, and outcome data) and evaluated the quality of included studies according to Cochrane Collaboration’s risk of bias tool in Cochrane Handbook for Systematic Reviews of Interventions 5.1.0. Consensus was used to resolve discrepancies. The end points of interest were: overall survival (OS), progression-free survival (PFS), no residual disease, residual disease ≤1 cm, and optimal cytoreduction rate. The definition of optimal cytoreductive surgery is residual tumor diameter ≤1 cm or no residual disease after debulking surgery.

### Statistical analysis

Pooled hazard ratios (HRs) for OS or PFS and relative risks (RRs) for extent of surgical debulking with corresponding 95% confidence interval (CI) were calculated in Review Manager(RevMan)^10^. We using the *I*^*2*^ statistic to assess statistical heterogeneity between studies^11^. An *I*^*2*^ ≤ 25%, 26% to 50%, and >50% indicates low, moderate, and high heterogeneity, respectively^12^. When *I*^*2*^ ≤ 25%, fixed effects model was presented^12^,^13^,^14^. On the contrary, random effects model was applied and sensitivity analysis was also carried out to investigating the influence of a single study on the overall pooled estimate by omitting one study in each turn. All statistical analysis were carried out by using RevMan 5.3.

## Results

### Literature search and study characteristics

**Figure 1:**
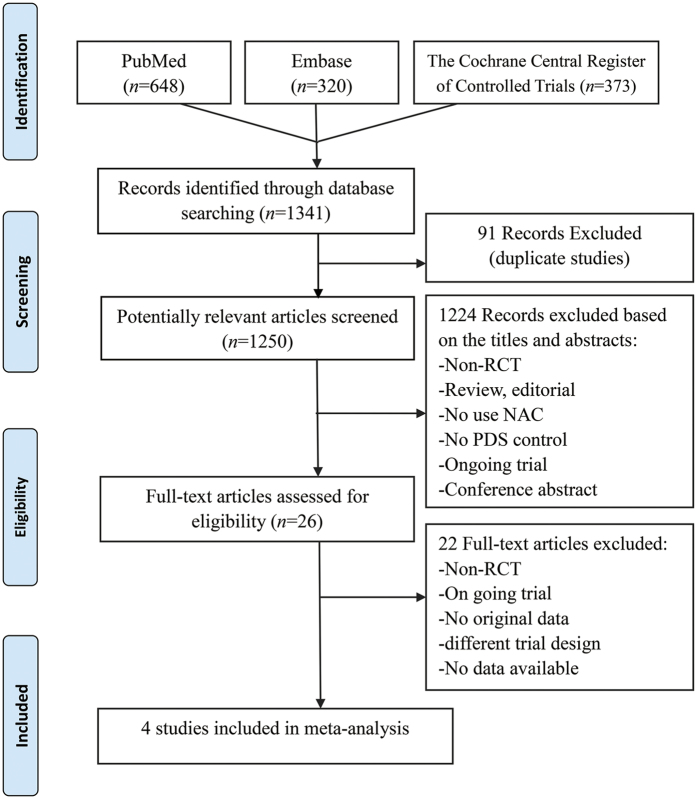
Flow diagram of literature search. RCT, randomized controlled trial.

The search process of the study is shown in Fig. 1. The initial screening yielded 1341 references. After the exclusion of duplicate publications, 1250 references were withheld for screening of the title and/or abstract, then 1224 references were excluded because of other reasons (non-RCT, reviews, editorials, no use of NAC, no PDS control, ongoing trials, and conference abstract). Finally after screening of the full article, only 4 RCTs containing 1922 patients were included in the meta-analysis process^1^,^2^,^4^,^5^. The major characteristics of the 4 RCTs included in the meta-analysis are presented in Table 1. The sample size of these trials ranged from 278 to 670 (total 1922 patients). The average age of the patients was similar between trials. All studies reported the OS and PFS^1^,^2^,^4^,^5^, and 2 studies compared optimal cytoreduction rate between both groups^1^,^2^.

**Figure 2:**
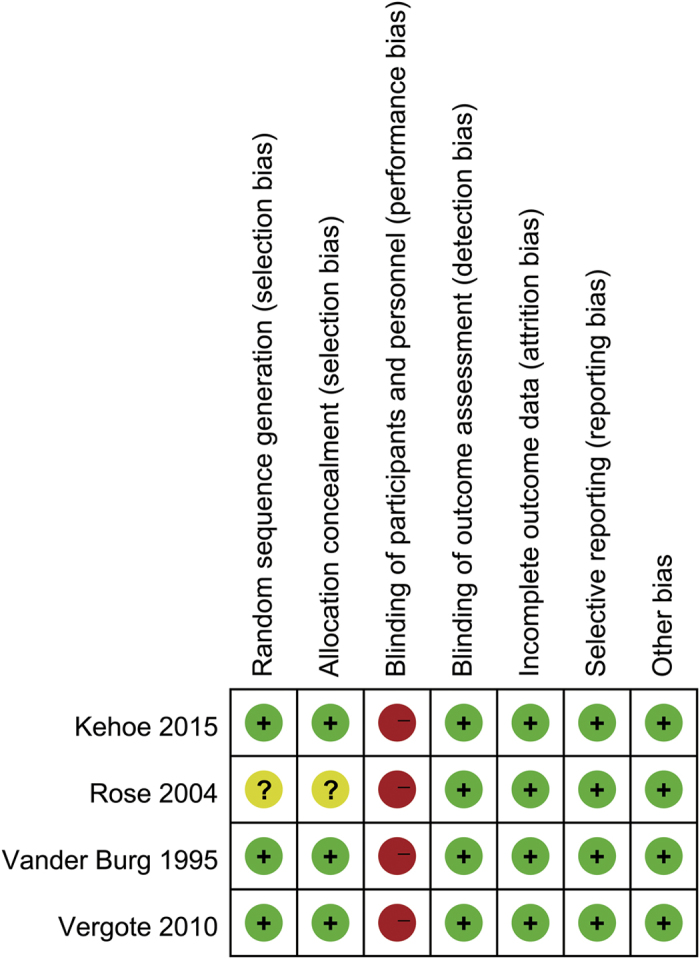
Assessment of quality of selected RCTs. Low risk of bias (green circles), unclear risk of bias (yellow circles) and high risk of bias (red circles).

The assessment of the quality of the selected RCTs is presented in Fig. 2. As less than 10 studies were included, we did not evaluate the publication bias in our study^15^.

### Overall survival

**Figure 3:**
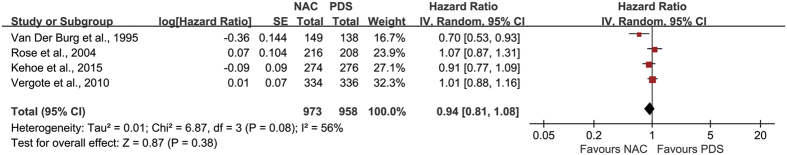
Forest plot for overall survival. NAC, neoadjuvant chemotherapy followed by interval debulking surgery; PDS, primary cytoreductive surgery followed by systemic chemotherapy.

All studies evaluated the OS between NAC group and PDS group^1^,^2^,^4^,^5^. The outcomes of OS were pooled and compared with a random-effects model (Fig. 3). There was no significant difference in OS between NAC group and PDS group (HR: 0.94; 95%Cl: 0.81–1.08; P = 0.38), with high heterogeneity among the studies (*I*^*2*^ = 56%). From the sensitivity analysis, we found that Van der Burg *et al*.^5^ probably contributed to the heterogeneity. After excluding this study, the result suggested that OS of AEOC patients was still similar between NAC and PDS (HR 0.99, 95%CI: 0.90–1.09, P = 0.90), with low heterogeneity (*I*^*2*^ = 0%).

### Progression-free survival

**Figure 4:**
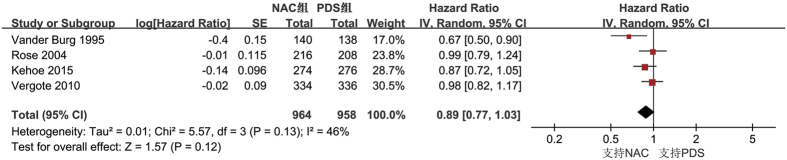
Forest plot for progression-free survival. NAC, neoadjuvant chemotherapy followed by interval debulking surgery; PDS, primary cytoreductive surgery followed by systemic chemotherapy.

All studies evaluated the PFS between NAC group and PDS group^1^,^2^,^4^,^5^. Pooling data from the 4 RCTs by a random-effects model included no significant difference in PFS was found between the NAC group and PDS group (HR: 0.89; 95% Cl: 0.77–1.03; P = 0.12), with moderate heterogeneity among the studies (*I*^*2*^ = 46%) (Fig. 4).

### Extent of surgical debulking

**Figure 5:**
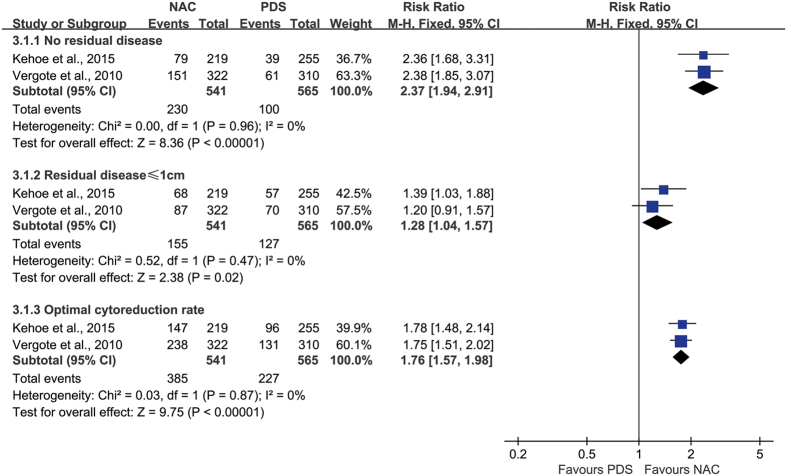
Forest plot for extent of surgical debulking. The definition of optimal cytoreductive surgery is residual tumor diameter ≤1 cm or no residual disease after debulking surgery; NAC, neoadjuvant chemotherapy followed by interval debulking surgery; PDS, primary cytoreductive surgery followed by systemic chemotherapy.

Two of the 4 studies reported data on the extent of surgical debulking, and thus were eligible to be included in the meta-analyses^1^,^2^. Compared with PDS, NAC may contribute to the completeness of lesions removal [no residual disease (RR: 2.37; 95%CI: 1.94–2.91; P < 0.00001; *I*^*2*^ = 0%), residual disease ≤1 cm (RR: 1.28; 95%CI: 1.04–1.57; P = 0.02; *I*^*2*^ = 0%), optimal cytoreduction rate (RR: 1.76; 95%CI: 1.57–1.98; P < 0.00001; *I*^*2*^ = 0%)] (Fig. 5).

## Discussion

We performed a meta-analysis of 4 RCTs to systematically evaluate the influence of NAC on survival and complete cytoreduction after debulking surgery in AEOC patients. In our meta-analysis, we found that NAC may contribute to the completeness of tumor removal [no residual disease (RR: 2.37; 95%CI: 1.94–2.91); residual disease ≤1 cm (RR: 1.28; 95%CI: 1.04–1.57); optimal cytoreduction rate (RR: 1.76; 95%CI: 1.57–1.98)], but OS and PFS of AEOC patients was similar between NAC and PDS [OS (HR: 0.94; 95%Cl: 0.81–1.08; P = 0.38); PFS (HR: 0.89; 95%Cl: 0.77–1.03; P = 0.12)]. Our findings are consistent with several previous meta-analysis which were based on retrospective cohort studies^8^,^9^.

According to the results of our analysis, NAC increased rate of optimal cytoreduction. It’s worth noting that Rose and colleagues (2004) state on that of 112 patients whose tumor exceeded 1 cm in diameter before they underwent secondary surgery, 79 (approximately 70%) had a residual mass of less than 1 cm and 33 (approximately 30%) had a residual mass of at least 1 cm after secondary surgery. The death rates in these two groups did not differ significantly (HR 1.25, 95% CI 0.785–2, p = 0.34). However, the studies by Rose *et al*.^4^ and van der Burg^5^ are fundamentally different in that all patients initially underwent a maximal cytoreductive effort complicated by inability to optimally debulk the cancer (interval debulking); this is in contrast to the more modern studies by Vergote and Kehoe in which patients underwent NO attempt at cytoreduction, only FNA/core biopsy often without laparotomy (or if laparotomy was performed, only biopsies were allowed) prior to randomization (pure neoadjuvant chemotherapy paradigm).

On the other hand, heterogeneity was noticed in OS between the included trials. From the sensitivity analysis, we found that the study conducted by Van der Burg *et al*. probably contributed to the heterogeneity^5^. Significantly different from other 3 studies, OS of NAC group was prolonged compared with PDS group in Van der Burg *et al*.5. A higher proportion of patients with large residual tumors in PDS group than the other 3 studies might have contributed to worse survival compared with the NAC group, and may thus be regarded as a potential source of heterogeneity^16^. Notably further sensitivity analysis excluding Van der Burg *et al*.^5^ did not appreciably alter our findings.

NAC has been controversial since its inception. Whether NAC improves the prognosis of AEOC has been the focus of much controversy. Optimal cytoreduction improves survival^17^, but the increase in optimal cytoreduction does not translate into a significant improvement of overall survival and progression-free survival in patients who receive NAC. In 2009, a meta-analysis^8^ of 21 non-randomized trials concluded that increased rate of optimal cytoreduction was found in NAC group, but survival was similar in both NAC and PDS groups^5^,^18^,^19^,^20^,^21^,^22^,^23^. In the retrospective setting, this may be due to better performance status and/or lesser extent of tumor in PDS group. In order to eliminate selection bias, the recent studies included RCTs only. Interestingly, we arrived at similar conclusions: NAC increased the rate of optimal cytoreduction, but did not provide equal survival compared with PDS. We hypothesize that chemotherapy before surgery might induce fibrosis, and the postoperative residual fibrosis tissue may contain cancer stem cells which promote chemotherapy resistance in AEOC patients who have received NAC^2^,^8^,^24^. More studies are needed to explore this mechanism. At a minimum, we can conclude that NAC was non-inferior to PDS.

With improvements in survival afforded by better therapies for original carcinoma, future studies should focus on quality of life (QoL). Recently, QoL outcome from final analysis of peri-operative outcome of Fagotti *et al*.^25^, using the EORTC quality of life questionnaire core-36 (QLQC-30) and the ovarian cancer-specific quality of life questionnaire (QLQ-Ov28), indicates QoL scores were shown to be more favorable in NAC group than PDS group in AEOC patients^26^,^27^. A similar trend towards better QoL was reported in Kehoe *et al*.^1^. More patients in NAC group reported improvement in QoL at least 5 points than PDS group at 6 and 12 months in Kehoe *et al*.^1^.

Which patients benefit most from NAC remains unknown. Due to poor performance status or older age of study population, unsatisfactory surgical outcomes were observed in PDS group of Vergote *et al*. and Kehoe *et al*.^1^,^2^. Though NAC is recommended for patients with bulky stage III/IV disease who are poor surgical candidates due to high-risk comorbidity conditions or disease factors by 2015 NCCN guideline, maximum surgical efforts and competent surgical skills are necessary, regardless of whether NAC is performed^28^.

Although we chose to include only RCTs in this study to minimize bias, there remain potential limitations of our meta-analysis. Due to different study designs, surgical techniques, chemotherapeutics regimens and surgical procedures, there was heterogeneity among the included trials. In order to mitigate the potential effect of heterogeneity on the validity of the results, we used a random-effects model and explored possible causes of heterogeneity by sensitivity analysis.

In summary, NAC is a reasonable treatment option for FIGO stage III and IV epithelial ovarian cancer patients with non-inferior survival compared with PDS. Furthermore emerging evidences suggests that NAC may be associated with improved QoL compared to PDS. Maximum surgical efforts and improvement of competent surgical skills are necessary, regardless of whether NAC is performed.

## Additional Information

**How to cite this article**: Zeng, L.-J. *et al*. Neoadjuvant chemotherapy for Patients with advanced epithelial ovarian cancer: A Meta-Analysis. *Sci. Rep.* **6**, 35914; doi: 10.1038/srep35914 (2016).

**Publisher’s note:** Springer Nature remains neutral with regard to jurisdictional claims in published maps and institutional affiliations.

## Figures and Tables

**Table 1:** The basic characteristics of included studies.

	Sample Size (Exp/Con)	Age (yr)^a^		Follow-up Time	FIGO Stage N (%)		Histopathologic Type,(Exp/Con)	Histologic Grade		Intervention	
		Exp	Con		Exp	Con		Exp	Con	Exp	Con
Kehoe *et al*.^1^	276/274	66	65		III:	III:	Serous 185/219	G1:13	G1:12	NAC × 3 cycles + IDS + Chemotherapy × 3cycles	PDS + chemotherapy × 6cycles
		(26–87)	(34–88)		206 (75.2%)	206 (74.5%)	Mucous 4/2	G2:43	G2:27		
					IV:	IV:	Clear cell 13/4	G3:165	G3:149		
					68 (24.8%)	70 (25.5%)	Endometrioid 5/11				
							Mixed 0/2				
							Unclassified 3/12				
Vergote *et al*.^2^	336/334	62	63		IIIC:	IIIC:	Serous 194/220	G1:14	G1:10	NAC × 3 cycles + IDS + Chemotherapy × 3cycles	PDS + chemotherapy × 6cycles
		(25–86)	(33–81)		253 (75.7%)	257 (76.5%)	Mucous 11/8	G2:57	G2:41		
					IV:	IV:	Clear cell 4/6	G3:145	G3:130		
					81 (24.3%)	77 (22.9%)	Endometrioid 5/11	Gx:120	Gx:153		
					other:	other:	Undifferentiated 90/69				
					0	2 (0.6%)	Mixed 0/9				
							Other/Unkoown 30/19				
Rose *et al*.^4^	208/216	57	58.1		III:	III:	Serous 165/159	G1:21	G1:19	PDS + NAC × 3 cycles + IDS + chemotherapy × 3cycles	PDS + chemotherapy × 6cycles
		(27.0–81.6)	(25.4–81.6)		200 (92.6%)	200 (96.2%)	Mucous 1/2	G2:82	G2:85		
					IV:	IV:	Clear cell 4/3	G3:105	G3:112		
					16 (7.4%)	8 (3.8%)	Endometrioid 17/11				
							Mixed 20/17				
							Undifferentiated/Other 5/8				
							Unspecified 4/8				
Van Der Burg *et al*.^5^	138/140	59	59		IIb:	IIb:	Serous 59/56	G1:9	G1:8	PDS + NAC × 3 cycles + IDS + chemotherapy × 3cycles	PDS + chemotherapy × 6cycles
		(32–74)	(32–74)		4 (4.1%)	4 (4.0%)	Mucous 8/4	G2:32	G2:27		
					III:	III:	Clear cell 1/4	G3:54	G3:61		
					71 (72.5%)	75 (75.0%)	Endometrioid 7/10	Gx:5	Gx:4		
					IV:	IV:	Mixed 0/0				
					23 (23.4%)	21 (21.0%)	Unclassified 25/26				

Exp, experimental group; Con, control group; NAC, neoadjuvant chemotherapy; PDS, primary debulking surgery;IDS, interval debulking surgery.

^a^Age: Median (Minimum age-Maximum age).
